# Activities of Ten Essential Oils towards *Propionibacterium acnes* and PC-3, A-549 and MCF-7 Cancer Cells

**DOI:** 10.3390/molecules15053200

**Published:** 2010-04-30

**Authors:** Yuangang Zu, Huimin Yu, Lu Liang, Yujie Fu, Thomas Efferth, Xia Liu, Nan Wu

**Affiliations:** 1 Key Laboratory of Forest Plant Ecology, Ministry of Education, Northeast Forestry University, Harbin 150040, China; E-Mails: zygorl@vip.hl.cn (Y.Z.); luliang20100224@yahoo.com.cn (L.L.); lx20030135@yahoo.com.cn (X.L.); bao_doubao@yahoo.com.cn (N.W.); 2 Engineering Research Center of Forest Bio-preparation, Ministry of Education, Northeast Forestry University, Harbin 150040, China; 3 Chinese Medicine Department, The Second Hospital of Harbin Medical University, Harbin 150086, China; E-Mail: huimin1973@126.com (H.Y.); 4 Department of Pharmaceutical Biology, Institute of Pharmacy, University of Mainz, 55099 Mainz, Germany; E-Mail: t.efferth@dkfz.de (T.E.)

**Keywords:** essential oils, activities, *Propionibacterium acnes*, cytotoxicity, cancer cell lines

## Abstract

Ten essential oils, namely, mint (*Mentha spicata* L*.*, Lamiaceae), ginger (*Zingiber officinale* Rosc*.*, Zingiberaceae), lemon (*Citrus limon *Burm.f*.*, Rutaceae), grapefruit (*Citrus paradisi *Macf., Rutaceae), jasmine (*Jasminum grandiflora* L*.*, Oleaceae), lavender (Mill., Lamiaceae), chamomile (*Matricaria chamomilla* L., Compositae), thyme (*Thymus vulgaris* L., Lamiaceae), rose (*Rosa damascena* Mill., Rosaceae) and cinnamon (*Cinnamomum zeylanicum* N. Lauraceae) were tested for their antibacterial activities towards *Propionibacterium acnes *and *in vitro* toxicology against three human cancer cell lines. Thyme, cinnamon and rose essential oils exhibited the best antibacterial activities towards *P. acnes*, with inhibition diameters of 40 ± 1.2 mm, 33.5 ± 1.5 mm and 16.5 ± 0.7 mm, and minimal inhibitory concentrations of 0.016% (v/v), 0.016% (v/v) and 0.031% (v/v), respectively. Time-kill dynamic procedures showed that thyme, cinnamon, rose, and lavender essential oils exhibited the strongest bactericidal activities at a concentration of 0.25% (v/v), and *P. acnes* was completely killed after 5 min. The thyme essential oil exhibited the strongest cytotoxicity towards three human cancer cells. Its inhibition concentration 50% (IC_50_) values on PC-3, A549 and MCF-7 tumor cell lines were 0.010% (v/v), 0.011% (v/v) and 0.030% (v/v), respectively. The cytotoxicity of 10 essential oils on human prostate carcinoma cell (PC-3) was significantly stronger than on human lung carcinoma (A549) and human breast cancer (MCF-7) cell lines.

## 1. Introduction

During recent years, plant essential oils have come more into the focus of phytomedicine [1,2]. Their widespread use has raised the interest of scientists in basic research of essential oils. Especially, the anti-microbial and anti-oxidant activities of essential oils as well as their potential anti-cancer activity have been investigated in recent years [3,4].

Acne is an inflammatory chronic disease, whose clinical presentation can range from a mild comedonal form to severe cystic acne of the face, chest, and back. Factors which contribute to the development of acne include hormonal imbalance, bacterial infection, stress, food, or cosmetic application [5]. *Propionibacterium acnes* is a Gram-positive, anaerobic microorganism, which has been most recognized as a key factor for the development of acne [6]. For many years antibiotics and hormones were usually applied to treat acne [7,8]. However, these agents are often accompanied by severe side effects and drug resistance [9,10]. Therefore, phytotherapeutic approaches with high anti-bacterial activity and without side effects have been extensively studied as an alternative. In this context, essential oils extracted from herbs have also been investigated for the treatment of acne [11]. 

Standard cancer chemotherapy is frequently compromised by the development of drug resistance and unwanted, partly life-threatening side effects. There is, therefore, an urgent need for novel treatment options with improved features. Interestingly many plant-derived compounds, *i.e.*, paclitaxel, vinblastine, or vincristine, and semi-synthetic derivatives of natural products, *i.e.*, etoposide and teniposide, are used as anti-cancer drugs. As pointed out recently, natural products from medicinal plants represent a fertile ground for the development of novel anticancer agents [12]. 

Interestingly essential oils from some herbs and spices possess both anti-bacterial and cancer chemopreventive activities [13]. In previous studies, 10 essential oils including mint, ginger, lemon, grapefruit, jasmine, lavender, chamomile, thyme, rose and cinnamon showed good pharmacological activity [14,15,16,17,18,19,20]. In the present study, the anti-bacterial activities of a panel of 10 essential oils towards *P. acnes *was investigated by the disc diffusion and broth dilution methods as well as detection of dynamic bactericidal processes. Furthermore, we analyzed the cytotoxicity of these 10 essential oils against the human cancer lines, A549, PC-3 and MCF-7, by means of the MTT (3-(4,5)-dimethylthiazol-2-yl)-2,5-diphenyltetrazolium bromide) assay. 

## 2. Results and Discussion

### 2.1. Anti-bacterial activity

**Figure 1:**
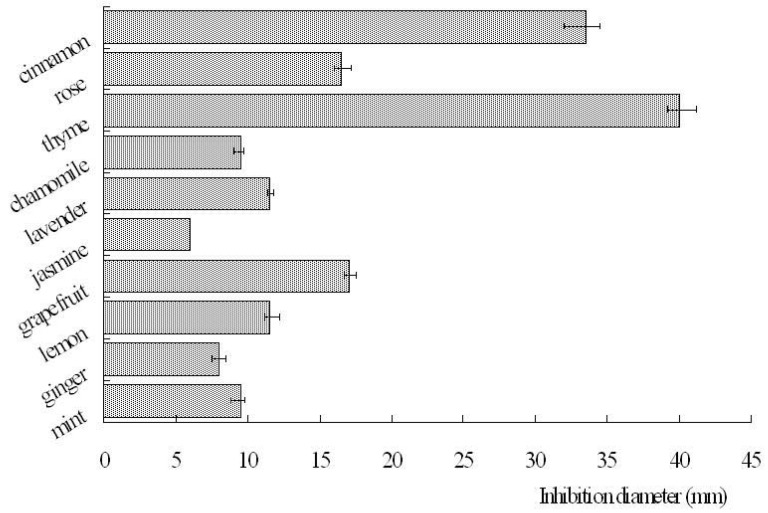
Inhibition diameters of 10 essential oils towards *P. acnes*.

We first analyzed the anti-bacterial activities of essential oils towards *P. acnes*. The data obtained from the disc diffusion method indicated that thyme essential oil exhibited the strongest inhibitory activities. The inhibition zone diameter measured was 40.0 ± 1.2 mm. Cinnamon essential oil also possessed considerable antibacterial activity, and the inhibition diameter determined was 33.5 ± 1.5 mm. Jasmine essential oil exhibited the lowest inhibitory activity (Figure 1). The results of minimum inhibitory concentrations (MICs) revealed that the thyme, cinnamon and rose essential oils exhibited the best anti-bacterial activities towards *P. acnes*. The MIC values were 0.016% (v/v), 0.016% (v/v) and 0.031% (v/v), respectively (Table 1). The anti-bacterial activity of jasmine essential oil was lower than that of other essential oils, and its minimum bactericidal concentration (MBC) was 0.5% (v/v). The MBC values of all 10 essential oils were comparable to their corresponding MIC values (Table 1).

**Table 1:** Minimal inhibitory concentrations (MICs, %v/v) and minimal bactericidal concentrations (MBCs, %v/v) of 10 essential oils towards *P. acnes*.

	cinnamon	rose	thyme	chamomile	lavender	jasmine	grapefruit	lemon	ginger	mint
MIC	0.016	0.031	0.016	0.125	0.125	0.500	0.250	0.250	0.250	0.250
MBC	0.016	0.031	0.016	0.125	0.125	0.500	0.250	0.250	0.250	0.250

### 2.2. Time-kill curves

**Figure 2:**
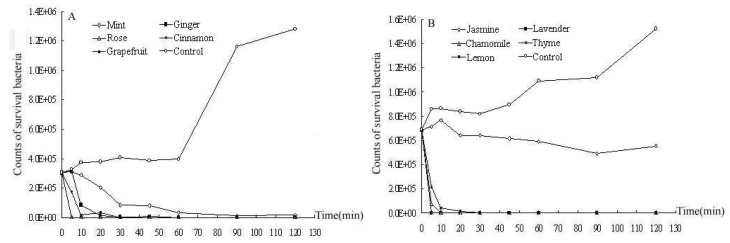
Time-kill curves of 10 essential oils (0.25% v/v) towards *P.acnes *in 2h. (A): essential oils of mint, ginger, rose, cinnamon, and grapefruit; (B): essential oils of jasmine, lavender, chamomile, thyme, and lemon.

The time-kill curves of the 10 essential oils are shown in Figure 2. Rose, cinnamon, thyme, and lavender essential oils exhibited the strongest bactericidal activities at a concentration of 0.25% (v/v); the bacteria were completely killed within 5 min. The bactericidal activities of the other essential oils decreased in the order: chamomile > grapefruit = lemon > ginger > mint > jasmine. The bacteria were completely killed after 20 min by chamomile essential oil, 30 min by grapefruit and lemon essential oils, and 45 min by ginger essential oil. However, mint essential oil did not kill bacteria even after 120 min. The jasmine essential oil exhibited the lowest bactericidal activity, and the number of *P.acnes* still retained 10^5 ^CFU/mL after 120 min. 

### 2.3. Cytotoxic activity towards cancer cells

**Figure 3:**
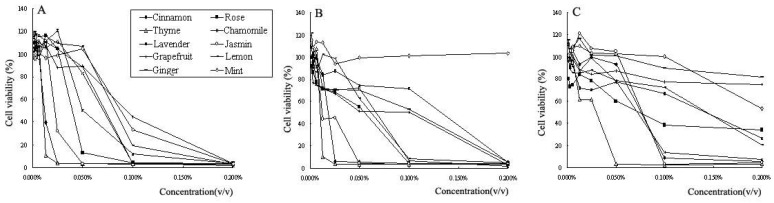
Dose-dependent cytotoxicity of 10 essential oils (72 h exposure) towards PC-3 (A), A549 (B) and MCF-7 (C) cell lines as determined by the MTT assay. Values are expressed as means ± SD of three independent experiments. Standard deviations were less than 15%.

To investigate the cytotoxic activities, three human tumor cell lines, A-549, PC- 3 and MCF-7, were exposed to increasing concentrations of essential oils. Cell viability was determined by the MTT assay. As shown in Figure 3 and Table 2 the essential oils revealed different cytotoxic activities towards the three human cancer cell lines investigated. In general, a dose-dependent decrease in the survival of the three tumor cell lines was observed. However, mint essential oil exhibited no effect on A549 cell over a concentration range of 0.002% to 0.2% (v/v). 

At a concentration of 0.002% (v/v), the essential oils did not considerably affect the viability of the three human tumor cell lines compared with untreated control cells. The cell survival after treatment with essential oils was more than 80%. 

At a concentration of 0.200% (v/v), however, all essential oils exhibited strong cytotoxicities towards PC-3 cells. Cell viability was lower than 4%. Most essential oils exhibited strong cytotoxicities towards A549 cells. However, cells treated with mint essential oil still grew well, and the number of survival cells was comparable to that of untreated control cells. For MCF-7 cell, the cytotoxictiies of cinnamon, thyme, chamomile, and jasmine essential oils was significantly stronger than that of the other six essential oils. The fractions of viable cells were reduced to 5.31%, 3.47%, 6.93% and 4.34%, respectively. Essential oils from grapefruit and ginger exhibited the lowest cytotoxicities towards MCF-7 cells. The percentages of cells viability were 75.03% and 81.85%, respectively. 

Of all essential oils investigated, thyme essential oil exhibited the strongest cytotoxicities towards cancer cells. The IC_50 _values for thyme essential oil against PC-3, A549 and MCF-7 cells were 0.010%, 0.011% and 0.030% (v/v), respectively. Moreover, cinnamon and jasmine essential oils possessed stronger cytotoxic activities towards PC-3 and A549 cell lines. IC_50_ values for cinnamon essential oil against PC-3 and A549 cells were 0.012% (v/v) and 0.017% (v/v). The IC_50_ values for jasmine essential oil against these two cell lines were 0.022% (v/v) and 0.012% (v/v) respectively. However, the IC_50_ values for cinnamon and jasmine essential oils against MCF-7 cells were 0.076% (v/v) and 0.077% (v/v). MCF-7 cell was less sensitive than the other cell lines towards the essential oils. 

**Table 2:** Inhibition concentrations 50% (IC_50, _%v/v) values for 10 essential oils of PC-3, A549 and MCF-7 cancer cell lines as determined by the MTT assay.

	cinnamon	rose	thyme	chamomile	lavender	jasmine	grapefruit	lemon	ginger	mint
PC-3	0.012	0.040	0.010	0.071	0.050	0.022	0.094	0.083	0.077	0.088
A549	0.017	0.055	0.011	0.067	0.133	0.012	0.100	0.061	0.107	-------
MCF-7	0.076	0.074	0.030	0.072	0.142	0.077	-------	0.143	-------	-------

Anti-bacterial and cytotoxic activities of essential oils can be attributed to their different constituents. Essential oils comprise complex mixtures, including monoterpenes and sesquiterpenes, such as limonene, menthol, α-pinene, 3-carene, and α-farnesol, etc. Some compositions have been reported for their anti-bacterial activities towards bacteria and fungi [21,22]. The anti-cancer activities of some monoterpenes and sesquiterpenes was also reported in the literature [23,24]. 

Until now, various authors have reported antitumor activities of essential oils as well as their components. For instance, the lavender essential oil was found to be active against COL-2 [25], the aldehyde compounds of *Citrus paradisi* essential oil induced apoptosis strongly in HL-60 cells [26], and thyme essential oil, which contains carvacrol, as the major component has an important *in vitro* cytotoxic activity against tumor cells [27]. In our results, ten essential oils (except for grapefruit, ginger and mint) also showed excellent antitumor activities against PC-3, A549, MCF-7. However, further studies are urgently needed for screening for the mechanism of the antitumor activity.

## 3. Experimental

### 3.1. Essential oils

Essential oils of mint (Mentha spicata), ginger (*Zingiber officinale*), lemon (Citrus limonum), grapefruit (*Citrus paradisi*), jasmine (Jasminum grandiflora), lavender (Lavandula stoechas), chamomile (Anthemis nobilis), thyme (Thymus vulgaris), rose (*Rosa centifolia*) and cinnamon (Cinnamomum zeylanicum) were obtained from a commercial source (Xiamen Denyla Essential Ooil Co., Ltd., Xiamen, China).

### 3.2. Maintenance of Proprionibacterium acnes

*P. acnes* (CMCC 65002) was purchased from China General Microbiological Culture Collection Center (CGMCC, Beijing, China). The organism was incubated in brain heart infusion medium (BHI) with 1% glucose (Aoboxing Biotech Company Ltd., Bejing, China) at 37 °C for 72 h under anaerobic conditions and adjusted concentration by direct microscopic counts before the assay.

### 3.3. Disc diffusion assay

The determination of inhibition diameters of *P. acnes* colonies by essential oils was carried out by the agar disc diffusion method [28]. The BHI agar media plate was swabbed with the bacterial suspension (10^8 ^CFU/mL) and kept for 30 min at 4 °C. Filter paper discs (6.0 mm in diameter) were soaked with 5 µL essential oil and placed on the surface of the inoculated BHI agar plates. Plates were incubated under anaerobic condition at 37 °C for 24 h. Three independent experiments were performed, and the diameters were recorded as mean values.

### 3.4. Determinition of MICs and MBCs values

The MIC (minimal inhibitory concentration) and MBC (minimal bactericidal concentration) tests were performed by the broth microdilution method [29]. The essential oils were dissolved in sterilized physiological saline solution (0.9%) supplemented with Tween-80 (Sigma) at final concentration of 0.5% (v/v). Serial two-fold dilutions from 1.000%–0.008% (v/v) of the essential oils were prepared and placed into a 96-well micro-titer plate. One hundred µL of sample of each concentration were dispensed into the wells of a micro-titer plate. Each well was then inoculated with 100 µL of the bacterial suspension. The final concentration of the suspension was adjusted to 10^5^ CFU/mL, and the plate was incubated under anaerobic condition at 37 °C for 24 h. After incubation, the wells were examined for growth of microorganisms and the MIC was determined. The MIC is defined as the lowest concentration of the essential oil at which the bacterium does not demonstrate visible growth. MBC was confirmed by reinoculating on agar plates with 10 µL of each culture medium from the microplates. The number of CFU/mL was determined after 24 h of incubation at 37 °C. MBC is defined as the lowest concentration of the essential oil at which incubated microorganisms are completely killed. Each experiment was repeated three times.

### 3.5. Time-kill dynamic curves

Time-kill dynamic procedures were performed as described by Avila *et al. *[30] with minor modifications. The final concentration of suspension of the strain was adjusted to 10^5^–10^6 ^CFU*/*mL. According to the results of MIC and MBC of 10 essential oils, the 0.25% (v/v) concentration of each essential oil was selected for use in the time-kill dynamic procedure. After incubating for 0, 5, 10, 20, 30, 45, 60, 90 and 120 min. with the broth micro dilution method, liquids (50 µL) were removed from the test solution for ten-fold serial dilution. Thereafter, a 25 µL liquid from each dilution was spread on the surface of the BHI agar plates and incubated at 37 °C under anaerobic condition for 24 h, and the number of CFU/mL was counted. The solution with no essential oil was used as a control. Experiments were carried out in triplicate. Time-kill curves were constructed by plotting the number of CFU/mL against time (min).

### 3.6. Maintenance of human cancer cell lines

Human lung carcinoma (A549), human prostate carcinoma (PC-3) and human breast cancer cell lines (MCF-7) were purchased from China Center for Type Culture Collection (Wuhan, China). These cell lines were grown and maintained in a humidified incubator at 37°C with a 5% CO_2_ atmosphere. Dulbecco’s modified Eagle’s medium (DMEM) supplemented with 10% fetal bovine serum (FBS), 100 U/mL penicillin and 100 µg/mL streptomycin was used for the A549 cell cultures. Roswell Park Memorial Institute Medium (RPMI) 1640 medium supplemented with 10% FBS, 100 U/mL penicillin and 100 µg/mL streptomycin was used as the culture medium for PC-3 and MCF-7 cells.

### 3.7. Cytotoxicity assay

The cytotoxic effects of the essential oils on three human tumor cell lines were assayed by the MTT assay [31]. The cells were seeded at a density of 5 × 10^4^ cells/well. The 10 essential oils were serially double diluted from 0.200% to 0.002% (v/v), and 100 μl liquid of each concentration was applied to the wells of a 96-well plate containing confluent cell monolayers (six wells per concentration). The dilution medium without the sample served as a control. After 72 h of incubation, MTT solution (5 mg/mL) was then added to each well. and the formazan precipitate was dissolved in 100 μL dimethyl sulfoxide after 4 h incubation, After shaking for 5 min, the content of the wells was homogenized on a microplate shaker. The optical densities (OD) were measured on a microplate ELISA reader at 570 nm. All tests and analyses were run in triplicate and mean values were recorded. The cell survival curves were calculated after comparing with the control. The percentage viability was calculated as follows:

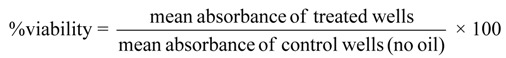



## 4. Conclusions

Among the 10 essential oils investigated in the present study, thyme and cinnamon essential oils exhibited the strongest anti-bacterial activities towards *P. acnes* and cytotoxic activities towards A549, PC-3, and MCF-7 human tumor cell lines. The main components of thyme essential oil is thymol [32], whose anti-microbial activities have been previously reported [33]. Its mechanism of action towards *S. aureus *and *E. coli* was also reported [34]. The main component of cinnamon is eugenol [35], which possesses notable anti-bacterial and anti-oxidant effects [36]. It was also found to be a potent inhibitor of melanoma cell proliferation [37]. We hypothesize that these two constituents may also be responsible for the antibacterial and cytotoxic activities of thyme or cinnamon essential oil observed in this investigation. Other essential oils also comprise monoterpenes, such as limonene and menthol, that possess bioactivity. On the basis of our results, 10 ten essential oils analyzed in this study may be used as alternative for food, cosmetics and medicine. In addition to their use for food and cosmetics, the potential of essential oils for the treatment of acne and cancer merits further exploration in the future.
